# Neuropsychological Measures of Attention and Impulse Control among 8-Year-Old Children Exposed Prenatally to Organochlorines

**DOI:** 10.1289/ehp.1104372

**Published:** 2012-02-22

**Authors:** Sharon K. Sagiv, Sally W. Thurston, David C. Bellinger, Larisa M. Altshul, Susan A. Korrick

**Affiliations:** 1 Department of Environmental Health, Boston University School of Public Health, Boston, Massachusetts, USA; 2 Channing Laboratory, Department of Medicine, Brigham and Women’s Hospital, Boston, Massachusetts, USA; 3 Department of Biostatistics and Computational Biology, University of Rochester School of Medicine and Dentistry, Rochester, New York, USA; 4 Children’s Hospital, Harvard Medical School, Boston, Massachusetts, USA; 5 Department of Environmental Health, Harvard School of Public Health, Boston, Massachusetts, USA; 6 Environmental Health and Engineering, Inc., Needham, Massachusetts, USA

**Keywords:** attention deficit hyperactivity disorder, *p*,*p*´-dichlorodiphenyl dichloroethylene (*p*,*p*´-DDE), epidemiology, maternal exposure, organochlorines, polychlorinated biphenyls (PCBs)

## Abstract

Background: We previously reported associations between organochlorines and behaviors related to attention deficit hyperactivity disorder among boys and girls at 8 years of age using a teacher’s rating scale for a birth cohort in New Bedford, Massachusetts (USA).

Objectives: Our goal was to corroborate these findings using neuropsychological measures of inattentive and impulsive behaviors.

Methods: We investigated the association between cord serum polychlorinated biphenyls (PCBs) and *p*,*p*´-dichlorodiphenyl dichloroethylene (*p*,*p*´-DDE) and attention and impulse control using a Continuous Performance Test (CPT) and components of the Wechsler Intelligence Scale for Children, 3rd edition (WISC-III). Participants came from a prospective cohort of children born during 1993–1998 to mothers residing near a PCB-contaminated harbor in New Bedford. Median (range) cord serum levels for the sum of four prevalent PCBs [congeners 118, 138, 153, and 180 (ΣPCB_4_)] and *p*,*p*´-DDE were 0.19 (0.01–2.59) and 0.31 (0–14.93) ng/g serum, respectively.

Results: We detected associations between PCBs and neuropsychological deficits for 578 and 584 children with CPT and WISC-III measures, respectively, but only among boys. For example, boys with higher exposure to ΣPCB_4_ had a higher rate of CPT errors of omission [rate ratio for the exposure interquartile range (IQR) = 1.12; 95% confidence interval (CI): 0.98, 1.27] and slower WISC-III Processing Speed (change in score for the IQR = –2.0; 95% CI: –3.5, –0.4). Weaker associations were found for *p*,*p*´-DDE. For girls, associations were in the opposite direction for the CPT and null for the WISC-III.

Conclusions: These results support an association between organochlorines (mainly PCBs) and neuropsychological measures of attention among boys only. Sex-specific effects should be considered in studies of organochlorines and neurodevelopment.

Organochlorines, including polychlorinated biphenyls (PCBs) and *p*,*p*´-dichlorodiphenyl dichloroethylene (*p*,*p*´-DDE), although banned in the United States since the 1970s, are still of public health importance because of their persistence in the environment and in human tissue, and because of the continued use of DDT (dichlorodiphenyltrichloroethane) in malaria-endemic countries (Schantz 1996; Van den Berg 2009). These lipophilic contaminants cross the placenta and affect fetal development, including neurodevelopment (Korrick and Sagiv 2008; Schantz et al. 2003).

Prenatal exposure to organochlorines, and to PCBs in particular, has been linked with behavioral impairments and functional deficits common to attention deficit/hyperactivity disorder (ADHD) (Eubig et al. 2010), the most common neurobehavioral disorder of childhood, affecting 5–10% of children worldwide (Faraone and Biederman 1998). PCBs have been reported to be associated with attention, response inhibition, and working memory, assessed primarily with neuropsychological tests (Grandjean et al. 2001; Jacobson et al. 1992; Stewart et al. 2000, 2003, 2005; Vreugdenhil et al. 2004). Animal data suggest sex differences in the effect of these exposures on neurodevelopment (Roegge et al. 2000; Schantz et al. 1995; Widholm et al. 2001); there are limited data for such differences in epidemiologic studies (Guo et al. 1995; Vreugdenhil et al. 2002).

We recently reported associations between prenatal PCB and *p*,*p*´-DDE levels and teacher-reported behaviors consistent with ADHD using a behavioral checklist (the Conners Rating Scale for Teachers) among 8-year-old children born to mothers residing adjacent to a PCB-contaminated harbor (Sagiv et al. 2010). We hypothesize an association between these persistent chemicals and standardized neuropsychological tests of attention and impulse control in this cohort.

## Methods

*Study population.* Children participating in this longitudinal cohort study were enrolled at birth. English- or Portuguese-speaking mothers ≥ 18 years of age residing in one of four towns (New Bedford, Acushnet, Fairhaven, Dartmouth) near a PCB-contaminated harbor in New Bedford, Massachusetts, for at least the duration of pregnancy were recruited from a local hospital with approximately 2,000 births per year. Approximately 10% of mothers met study eligibility criteria and were available for recruitment during times when a study examiner was on site. Infants too ill to undergo neonatal examination or born by cesarean section were excluded from the study. Of the 788 mother–infant pairs enrolled at birth, 607 were followed up for neurodevelopmental testing when the child was approximately 8 years of age (78% of those eligible). Multiple births (*n* = 3 children) were excluded from the present analysis.

*Exposure assessment.* Cord blood samples for organochlorine analyses were collected at the infant’s birth; the serum fraction was removed after centrifugation and stored at –20°C. All sample analyses were performed by the Harvard School of Public Health Organic Chemistry Laboratory (Boston, MA). Laboratory personnel were blinded to health outcomes. Cord serum samples were analyzed for 51 individual PCB congeners and *p*,*p*´-DDE. Laboratory analytic methods and quality control procedures are described elsewhere (Korrick et al. 2000). Briefly, liquid-liquid extraction and column chromatography cleanup were used, and the extracts were analyzed by gas chromatography with electron capture detection using an internal standard. Primary and confirmatory capillary columns were used, and where results differed, the lower value was reported.

PCB concentrations were reported as individual congeners in units of nanograms per gram serum after the amount of analyte in the procedural blank was subtracted. Lipid content could not be determined for study subjects because of insufficient sample volume (1.5–4 mL) and was therefore measured for 12 randomly selected cord bloods from discarded, anonymous samples collected at the study recruitment site; values were reproducible (1.7 g/L ± 0.3).

The method detection limits (MDLs) for individual PCBs ranged from 0.002 to 0.04 ng/g serum, with most MDLs < 0.01 ng/g; the MDL for *p*,*p*´-DDE in serum was 0.07 ng/g (Korrick et al. 2000). The frequency of values below the MDL for each congener for this study cohort was previously reported (Korrick et al. 2000). We retained measured values below the detection limit to optimize statistical power and avoid biased exposure estimates associated with censoring at the MDL (Kim et al. 1995). Reproducibility of serum analyses was good; the within-batch coefficient of variation (CV) for the sum of four prevalent PCB congeners [118, 138, 153, and 180 (ΣPCB_4_)] was 3% and the between-batch CV was 20% over the 5 years of analysis, with similar performance for *p*,*p*´-DDE.

*Outcome assessment.* We analyzed components of a continuous performance test (CPT) and a psychometric test of intelligence that reflect attention and impulse control; both tests were administered at the 8-year exam. The Neurobehavioral Examination System 2 (NES2) CPT is a computer-assisted examination that measures response to visual cues in a continuous performance task (Letz 1998). A random series of animal silhouettes are displayed on the screen, and the child is instructed to press a button on a joystick only upon the appearance of a cat, to respond as quickly as possible, and to refrain from pressing the button for any other animal. The computer records response time (recorded only for correct responses), the number of nonresponses or errors of omission (button not pressed when cat appeared; the maximum time given was 1,200 msec), and the number of false-positive errors or errors of commission (button pressed for an animal other than the cat). Four blocks of trials were completed, with the first block designated as a practice block that was not scored. We analyzed four components of the CPT: *a*) mean response time, *b*) response time variability (standard deviation of mean response time), *c*) total errors of omission, and *d*) total errors of commission. Outcomes were summed across the last three test blocks. Inattention was interpreted as a higher number of errors of omission and longer reaction time, and poor response inhibition was interpreted as a higher number of errors of commission. Higher reaction time variability or performance inconsistency is also thought to indicate fluctuations or lapses in attention (Van de Molen 1996).

The Wechsler Intelligence Scale for Children, 3rd edition (WISC-III), is a test that evaluates intellectual abilities (Wechsler 1991). We focused on the two specific age-standardized subscales for which children with ADHD are found to score lowest: Processing Speed (includes coding and symbol search)and Freedom from Distractibility (includes digit span and arithmetic) (Wechsler 1991).

*Statistical analysis.* We analyzed associations between attention and impulse control and two PCB congener groups: *a*) ΣPCB_4_, the sum of four prevalent PCB congeners with relatively high levels that were measured with less error, and *b*) the computed toxic equivalent (TEQ) for the sum of the five dioxin-like mono-*ortho* PCB congeners measured (105, 118, 156, 167, and 189), computed on a lipid basis (1.7 g/L) and weighted with toxic equivalency factors (Van den Berg et al. 2006). The TEQ group was included to investigate the potential for an aryl hydrocarbon receptor–mediated mechanism for the effect of dioxin-like congeners on neurodevelopment. We also investigated associations with *p*,*p*´-DDE.

CPT mean reaction time and reaction time variability and WISC-III outcomes were approximately normally distributed, met regression model assumptions, and were modeled with linear regression. Processing Speed and Freedom from Distractibility scores were standardized (mean ± SD = 100 ± 15). CPT errors of omission and commission were considered count data and initially modeled as Poisson distributed variables with log risk models; to correct for overdispersion (variance exceeded the mean), our final models were fitted using negative binomial regression.

Covariate data came from multiple sources, including maternal and pediatric medical records (including lead screening) and questionnaires administered 2 weeks after birth and at the 8-year follow-up examiantion. The 8-year exam included an assessment of maternal intelligence and depression using the Kaufman Brief Intelligence Test (Kaufman and Kaufman 1990) and the Beck Depression Inventory (Beck et al. 1996), respectively, and family and home characteristics using the Home Observation for Measurement of the Environment (HOME) (Caldwell and Bradley 1984). Potential confounders considered were characteristics of the mother [age at child’s birth, prenatal smoking and alcohol consumption, prenatal diet (local and overall fish consumption), illicit drug use in the year before the child’s birth, breast-feeding, parity, and, at the 8-year examination, education, marital status, IQ, and depression] and the child (age at exam, birth year, sex, race/ethnicity, ADHD medication use, and peak and mean 12- to 36-month blood lead levels). Examiner (there were two possible examiners), household income, and quality of the home environment (HOME score) assessed at the time of the 8-year examination were also considered.

Inclusion of covariates in multivariable models was based on *a priori* considerations of covariate associations with exposure and outcome, model fit (statistically significant partial *F*-test at α < 0.10), and whether covariate inclusion materially affected the organochlorine exposure effect estimate. To check the sensitivity of our estimates to covariates that were not included in the model, we added each back to the final model to make sure they did not substantially change our final effect estimates. We also assessed differences in exposure–outcome associations across sex using stratified analyses and by including an interaction term between sex and exposure in the model.

Based on recent literature suggesting that performance on the CPT may vary over the course of the test session (Julvez et al. 2010) (scores averaged over the entire testing period could mask these differences), block-specific effect estimates were also investigated. The Julvez et al. (2010) paradigm divides performance over a 10-min testing period into three sequential stages: *a*) orientation, learning, and habituation (first 2 min), *b*) processing speed and selective focused attention (next 4 min), and *c*) sustained attention (last 4 min). Because our children were younger than those in the Julvez et al. (2010) study, we used a 4-min CPT, where block 1 is omitted as a training block and blocks 2–4 (1 min each) correspond to Julvez’s three sequential stages. We investigated block-specific results for all four CPT outcomes.

Sensitivity of our results to ADHD medication use was explored by excluding children with parent-reported medication use and recomputing organochlorine exposure–outcome associations. In addition, we investigated the influence of missing covariate data on our results by comparing unadjusted exposure–outcome associations for all children (regardless of whether covariate data was missing) with unadjusted exposure–outcome associations in the subset of children with nonmissing covariate data.

The study protocol was reviewed and approved by the Human Subjects Committees of Harvard School of Public Health and Brigham and Women’s Hospital (Boston, MA) and Southcoast Hospitals Group (New Bedford, MA). Written informed consent was obtained from all participating families before study evaluations.

## Results

Exposure and outcome summary statistics are displayed in Table 1 for children administered the CPT and WISC-III exam who also had exposure data. An observation with an extremely high PCB value (ΣPCB_4_ > 4 ng/g) that disproportionately influenced exposure–outcome associations was excluded from all analyses. Approximately half of the total PCB concentration (sum of 51 congeners, mean ± SD = 0.53 ± 0.55 ng/g serum) was attributable to ΣPCB_4_ (mean ± SD = 0.25 ± 0.26 ng/g serum). PCBs and *p*,*p*´-DDE were positively correlated, with Spearman correlation coefficients ranging from 0.56 (mono-*ortho* PCB TEQ and *p*,*p*´-DDE) to 0.88 (mono-*ortho* PCB TEQ and ΣPCB_4_).

**Table 1:** Summary statistics for cord serum organochlorines and CPT and WISC-III outcomes for children with both exposure and outcome measures born in New Bedford, 1993–1998.

Exposure or outcome measure	n	Mean ± SD	Median (range)	IQR
Exposure statistics								
ΣPCB4 (ng/g)		584		0.25 ± 0.26		0.19 (0.01–2.59)		0.19
PCB TEQ (pg/g lipid)		584		1.41 ± 1.97		0.89 (0.00–26.56)		1.00
p,p´-DDE (ng/g)		584		0.50 ± 1.03		0.31 (0.00–14.93)		0.27
Outcome statistics								
CPT (n = 578)								
Mean reaction time (msec)		578		646.7 ± 65.0		645.4 (487.8–861.7)		
Reaction time variability (msec)		578		127.7 ± 31.7		125.9 (53.2–393.9)		
Total errors of omission		578		2.3 ± 2.7		1.0 (0.0–17.0)		
Total errors of commission		578		2.4 ± 2.2		2.0 (0.0–14.0)		
WISC-III, age-standardized scores (n = 584)								
Processing Speed		584		104.5 ± 14.8		104.0 (58.0–146.0)		
Freedom from Distractibility		583		98.1 ± 13.1		98.0 (50.0–134.0)		

Supplemental Material, Tables 1 and 2 (http://dx.doi.org/10.1289/ehp.1104372), presents the distribution of background characteristics and their unadjusted associations with the CPT and WISC-III outcomes. Briefly, 11% and 24% of mothers and fathers, respectively, did not complete high school; 20% had an annual household income < $20,000/year, and 41% were unmarried at the child’s 8-year examination, representing a socioeconomically diverse population. In addition, 31% of children were of nonwhite race/ethnicity, with 11% of Cape Verdean ethnicity, which is representative of the New Bedford population.

**Table 2:** Associations between ΣPCB4, PCB TEQ, and p,p´-DDE and CPT and WISC-III outcomes for all children and by sex for 8-year-old children born in New Bedford, 1993–1998 [IQRa (95% CI)].

Unadjusted model (all children)	Adjusted modelb					Sex interaction p-valuec
	Outcome measure	All children	Females	Males		
CPT		n = 578		n = 512		n = 254		n = 258		
Reaction time (β)										
ΣPCB4		–3.2 (–7.0, 0.6)		–3.1 (–7.3, 1.0)		–5.8 (–10.5, –1.0)		3.5 (–3.6, 10.5)		0.02
PCB TEQ		–2.8 (–5.4, –0.1)		–2.1 (–4.9, 0.7)		–3.4 (–6.5, –0.4)		3.0 (–2.6, 8.6)		0.04
p,p´-DDE		–0.7 (–2.0, 0.7)		–0.6 (–1.9, 0.8)		–1.5 (–3.0, 0.1)		1.8 (–0.7, 4.2)		0.03
Reaction time variability (β)										
ΣPCB4		–0.5 (–2.3, 1.4)		0.3 (–1.6, 2.2)		–0.7 (–2.9, 1.4)		2.9 (–0.3, 6.2)		0.05
PCB TEQ		–0.4 (–1.7, 0.9)		0.4 (–0.9, 1.7)		–0.4 (–1.8, 1.0)		3.3 (0.7, 5.8)		0.01
p,p´-DDE		–0.2 (–0.9, 0.4)		–0.1 (–0.7, 0.6)		–0.4 (–1.1, 0.3)		0.8 (–0.4, 1.9)		0.09
Errors of omission (rate ratio)										
ΣPCB4		1.00 (0.93, 1.07)		1.01 (0.93, 1.10)		0.95 (0.86, 1.05)		1.11 (0.98, 1.27)		0.04
PCB TEQ		1.00 (0.95, 1.06)		1.02 (0.97, 1.08)		0.98 (0.92, 1.05)		1.12 (1.01, 1.25)		0.03
p,p´-DDE		1.00 (0.97, 1.03)		1.00 (0.97, 1.03)		0.97 (0.94, 1.01)		1.05 (0.99, 1.10)		0.02
Errors of commission (rate ratio)										
ΣPCB4		1.00 (0.95, 1.06)		1.01 (0.95, 1.07)		1.00 (0.93, 1.08)		1.03 (0.93, 1.13)		0.68
PCB TEQ		1.00 (0.97, 1.04)		1.02 (0.98, 1.06)		1.01 (0.97, 1.06)		1.04 (0.96, 1.12)		0.63
p,p´-DDE		1.00 (0.98, 1.02)		1.01 (0.99, 1.03)		1.00 (0.98, 1.02)		1.02 (0.99, 1.06)		0.23
WISC-III		n = 584		n = 535		n = 264		n = 271		
Processing Speed (β)										
ΣPCB4		0.3 (–0.6, 1.2)		–0.5 (–1.5, 0.4)		0.0 (–1.0, 1.1)		–2.0 (–3.5, –0.4)		0.03
PCB TEQ		0.3 (–0.3, 0.9)		–0.1 (–0.7, 0.5)		0.2 (–0.4, 0.9)		–1.4 (–2.7, –0.2)		0.02
p,p´-DDE		0.0 (–0.4, 0.3)		–0.2 (–0.5, 0.1)		0.0 (–0.4, 0.3)		–0.4 (–1.0, 0.1)		0.22
Freedom from Distractibility (β)d										
ΣPCB4		0.2 (–0.6, 0.9)		–0.1 (–0.9, 0.7)		–0.1 (–1.0, 0.8)		–0.2 (–1.6, 1.2)		0.91
PCB TEQ		0.0 (–0.5, 0.6)		0.0 (–0.6, 0.5)		0.0 (–0.6, 0.6)		–0.2 (–1.4, 0.9)		0.68
p,p´-DDE		–0.1 (–0.4, 0.2)		–0.1 (–0.3, 0.2)		–0.2 (–0.5, 0.2)		0.1 (–0.4, 0.6)		0.40
Sex-specific estimates are from a single model (total n = x). aBeta (linear regression model) or rate ratio (negative binomial regression model) for an IQR (ΣPCB4 = 0.19 ng/g, PCB TEQ = 1.00 pg/g lipid, p,p´-DDE = 0.27 ng/g) increase in exposure. bCPT models were adjusted for child’s age at examination, sex, and birth year; maternal age at birth and prenatal smoking; and maternal and paternal education. WISC-III models were adjusted for child’s age at examination and sex and maternal age at birth, prenatal smoking, intelligence, and education; a higher WISC-III score indicates better performance. cSex interaction p-value is based on a likelihood ratio test. dFor Freedom from Distractibility, unadjusted n = 583.										

Unadjusted and adjusted associations between CPT and WISC-III outcomes and organochlorines, including the ΣPCB_4_, mono-*ortho* PCB TEQ, and *p*,*p*´-DDE, for all children (males and females combined) were very weak or null (Table 2). Furthermore, CPT mean reaction time was slightly shorter with greater exposure, in contrast to the hypothesized direction for this association.

**Figure 1:**
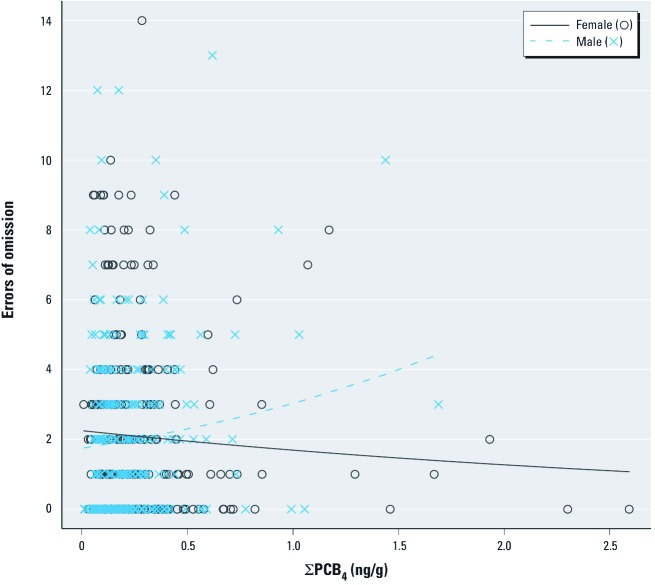
Unadjusted scatterplots and adjusted negative binomial regression lines of the association between cord serum levels of ΣPCB_4 _and errors of omission on the CPT by sex for 8-year-old children born in New Bedford, 1993–1998, adjusted for child’s age at examination, sex, and birth year; maternal age at birth and prenatal smoking; and maternal and paternal education. Adjusted rate ratios: females, 0.95 (95% CI: 0.86, 1.05); males, 1.11 (95% CI: 0.98, 1.27).

Results were notably different by sex (Table 2). Although females had considerably shorter CPT mean reaction time with increasing exposure, particularly for the ΣPCB_4_ [for an interquartile range (IQR) increase in ΣPCB_4_: β = –5.8; 95% confidence interval (CI): –10.5, –1.0], the opposite association was observed for males, with longer mean reaction time with increasing exposure (for an IQR increase in ΣPCB_4_: β = 3.5; 95% CI: –3.6, 10.5); the ΣPCB_4_ × sex interaction was statistically significant using a likelihood ratio test (*p* = 0.02). Reaction time variability also differed by sex with lower variability among females and higher variability in males with increasing exposure to ΣPCB_4_, mono-*ortho* PCB TEQ, and *p*,*p*´-DDE. PCB-associated omission error rate was also higher among males than among females, with an 11% increase in the rate of an error of omission per IQR increase in ΣPCB_4_ among males and no association among females (*p*-value for interaction = 0.04). This pattern is illustrated in Figure 1, where a higher rate of errors of omission was observed with higher ΣPCB_4_ levels among males and the opposite association was observed for females. Errors of commission were not associated with organochlorines in males or females (Table 2). In general, associations with *p*,*p*´-DDE were weaker than those with ΣPCB_4_ or mono-*ortho* PCB TEQ.

ΣPCB_4_–CPT associations were further explored by block period (Table 3). Associations among males were stronger for the third and fourth block periods than for the second block period for most CPT outcomes. Block-specific trends among females were less consistent. Results were similar for PCB TEQ (data not shown).

**Table 3:** Association between ΣPCB4 (ng/g) and CPT measures of attention and impulse control by block period for all children and by sex for 8-year-old children born in New Bedford, 1993–1998: adjusted modela [IQRb (95% CI)].

Outcome measure	All children (n = 512)	Females (n = 254)	Males (n = 258)	Sex interaction p-valuec
Reaction time (β)								
Block 2		–4.1 (–8.8, 0.7)		–6.5 (–11.9, –1.1)		2.0 (–6.0, 10.1)		0.07
Block 3		–2.5 (–7.2, 2.2)		–5.4 (–10.7, 0.0)		4.5 (–3.5, 12.4)		0.03
Block 4		–2.7 (–7.3, 2.0)		–5.3 (–10.7, 0.0)		3.9 (–4.0, 11.8)		0.05
Reaction time variability (β)								
Block 2		–0.3 (–3.0, 2.3)		–1.3 (–4.3, 1.7)		2.0 (–2.5, 6.5)		0.21
Block 3		1.1 (–1.7, 3.8)		0.3 (–2.8, 3.5)		2.9 (–1.8, 7.5)		0.36
Block 4		0.0 (–2.8, 2.8)		–1.4 (–4.7, 1.8)		3.6 (–1.2, 8.4)		0.07
Errors of omission (rate ratio)								
Block 2		1.00 (0.90, 1.12)		0.95 (0.82, 1.10)		1.08 (0.91, 1.27)		0.23
Block 3		1.04 (0.94, 1.14)		0.98 (0.87, 1.10)		1.15 (0.99, 1.33)		0.07
Block 4		0.98 (0.88, 1.09)		0.90 (0.78, 1.05)		1.08 (0.93, 1.26)		0.07
Errors of commission (rate ratio)								
Block 2		1.00 (0.92, 1.07)		1.03 (0.94, 1.12)		0.94 (0.83, 1.06)		0.21
Block 3		0.97 (0.87, 1.08)		0.85 (0.72, 1.01)		1.10 (0.95, 1.27)		0.01
Block 4		1.05 (0.96, 1.14)		1.05 (0.94, 1.16)		1.05 (0.91, 1.20)		0.98
Sex-specific estimates are from a single model (total n = x). aAdjusted for child’s age at examination, sex, and birth year; maternal age at birth and prenatal smoking; and maternal and paternal education. bBeta or rate ratio for an IQR increase in exposure. cSex interaction p-value is based on a likelihood ratio test.								

**Figure 2:**
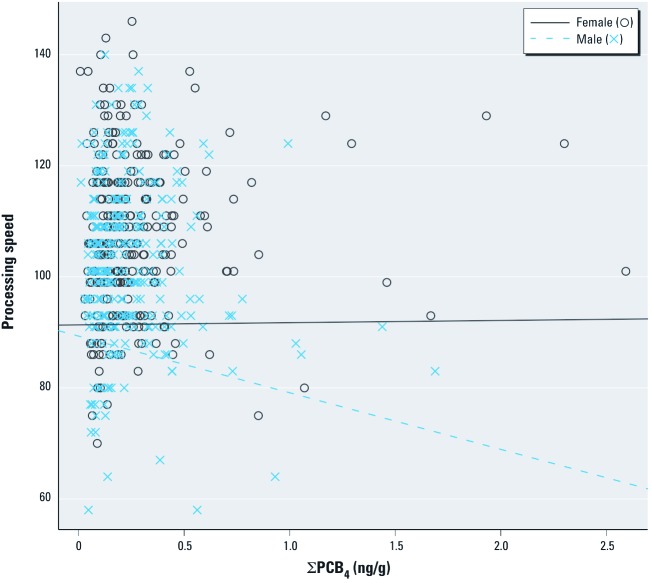
Unadjusted scatterplots and adjusted linear regression lines of the association between cord serum levels of ΣPCB_4 _and processing speed on the WISC-III by sex for 8-year-old children born in New Bedford, 1993–1998, adjusted for child’s age at examination and sex, maternal age at birth and prenatal smoking, and maternal intelligence and maternal education. Adjusted β-values: females, 0.0 (95% CI: –1.0, 1.1); males, –2.0 (95% CI: –3.5, –0.4).

Sex-specific effects were also detected between organochlorines and WISC-III Processing Speed (Table 2). Males had lower scores, indicating slower Processing Speed, with higher exposure (for an IQR increase in ΣPCB_4_: β = –2.0; 95% CI: –3.5, –0.4), whereas associations for females were null (*p*-value for interaction = 0.03), as illustrated in Figure 2. A similar pattern of associations was observed for PCB TEQ (Table 2). All associations were null for Freedom from Distractibility, and all *p*,*p*´-DDE–WISC-III associations were null.

Sensitivity analysis (data not shown) excluding children with a history of ADHD medication use (*n* = 45) did not change exposure–outcome associations. In addition, lead was not a confounder of the association between organochlorines and CPT or WISC-III measures reported here, which is consistent with the absence of an association between lead and organochlorines in our study (e.g., 2-year blood lead Spearman correlation coefficients with ΣPCB_4_, mono-*ortho* PCB TEQ, and *p*,*p*´-DDE were –0.08, –0.07, and –0.05, respectively). Finally, the unadjusted exposure effects were essentially unchanged when restricted to children with nonmissing covariate data (CPT, *n* = 512; WISC-III, *n* = 535), suggesting that excluding data from subjects with missing covariates data did not bias our estimates.

## Discussion

Cord serum PCB levels were low for this cohort relative to other population-based studies (Korrick et al. 2000; Longnecker et al. 2003), given maternal residence adjacent to the PCB-contaminated New Bedford Harbor. Despite low exposure levels, among males we found evidence for an association between organochlorines, especially PCBs, and neuropsychological end points, measured with the NES2 CPT and WISC-III. Associations, albeit modest, were strongest for CPT errors of omission and reaction time variability, indicators of inattention, and WISC-III Processing Speed, also related to attentional skills. Organochlorines were also associated with longer reaction time among males, but CIs were wide. Among females, associations were generally null, except for mean reaction time, which was shorter with higher organochlorine exposure, the opposite of the hypothesized direction of effect. This unexpected finding among girls could have been attributable to chance or residual confounding.

We previously reported moderate associations of cord serum PCB and *p*,*p*´-DDE levels with inattentive and hyperactive-impulsive behaviors assessed with the Conners Rating Scale for Teachers at approximately 8 years of age in the same cohort (Sagiv et al. 2010). Although not reported in that article, we did not detect sex differences in the association between organochlorines and teacher-rated behavior.

Several other studies report associations between PCBs and attention/impulse control using CPTs. Slower reaction time was observed in three studies in relation to prenatal PCB exposure (Grandjean et al. 2001; Jacobson et al. 1992; Vreugdenhil et al. 2004). A prospective birth cohort in Michigan found associations between cord serum PCBs and slower reaction time on a visual discrimination task at 4 years of age; however, associations with reaction time were null for a CPT administered at 11 years of age in the same study population (Jacobson and Jacobson 2003; Jacobson et al. 1992). Associations between late pregnancy maternal serum PCBs and slower reaction time on the Simple Reaction Time Test at 9 years of age were also observed in a cohort in the Netherlands (Vreugdenhil et al. 2004). A study conducted in the Faroe Islands found associations between cord tissue PCB levels and longer reaction time on the NES2 CPT at 7 years of age (the same test used in the present study), although only in the presence of high cord blood mercury (Grandjean et al. 2001).

Two studies have reported associations between PCBs and errors of commission, an indicator of response inhibition, on the CPT (Jacobson and Jacobson 2003; Jacobson et al. 1992; Stewart et al. 2003, 2005). The Michigan cohort detected more frequent errors of commission on the Sternberg visual and recognition memory test at 4 years of age and on the Catch-the-Cat CPT at 11 years of age in relation to prenatal PCB exposure (Jacobson and Jacobson 2003; Jacobson et al. 1992). Participants in the Oswego Children’s Study with higher cord serum PCB levels also made more errors of commission on the Michigan Catch-the-Cat CPT at 4 years of age (Stewart et al. 2003), the NES2 CPT at 8 years of age, and an extended CPT, which was designed specifically to differentiate between response inhibition and sustained attention by varying the target presentation rate, at 9 years of age (Stewart et al. 2005). An association between PCBs and impaired response inhibition in this cohort was further supported using a delayed reinforcement paradigm at 9 years of age (Stewart et al. 2006).

In contrast to the Michigan and Oswego study findings, associations between ΣPCB_4_, PCB TEQ, and *p*,*p*´-DDE and errors of commission were null in the present study. Instead, we found associations between PCBs/*p*,*p*´-DDE and CPT errors of omission, an indicator of inattention, among males. One possible explanation for different sensitivity to errors of commission versus omission is the low target presentation rate in the NES2 CPT; that is, the target is presented infrequently. Results from Stewart et al. (2005) based on the Extended CPT suggest that PCB associations with response inhibition are stronger using a test with a high target presentation rate. The NES2 CPT used in our study has a low target presentation rate and is therefore more suited for detecting inattention, demonstrated by more frequent errors of omission which could explain our ability to detect this behavior, compared with response inhibition.

Stronger effects of PCBs on CPT outcomes were detected for later blocks (third and fourth blocks, compared with second block) primarily for males. These findings were consistent with a previous study of methylmercury and NES2 CPT reaction time (Julvez et al. 2010), which attributed this trend to higher neurotoxicant sensitivity of sustained attention (and, for this study, perhaps focused attention) represented by these later block periods.

Our study also found associations between PCBs and Processing Speed, a subscale of the WISC-III, among males only. Although associations between PCBs and Processing Speed have not been previously reported, associations were found with Freedom from Distractibility, another WISC-III subscale, in the Michigan study (Jacobson and Jacobson 1996), with suggestive associations for Digit Span, a subtest of Freedom from Distractibility (Jacobson and Jacobson 2003). Null associations were found between PCBs and Digit Span in the Faroes cohort (Grandjean et al. 2001) and the present study (data not shown).

Sex differences in estimated PCB effects were not found for CPT outcomes in the Oswego study (Stewart et al. 2005) and were not reported for the Michigan CPT study (Jacobson and Jacobson 2003). However, sex differences in organochlorine associations with neurodevelopment have been observed in other studies. Among the Yu-Cheng cohort, a Taiwanese population heavily exposed prenatally (or via lactation) to cooking oil contaminated with PCBs and polychlorinated dibenzofurans, decreased cognitive function was observed among exposed males but not females (Guo et al. 1995). Sex differences in prenatal PCB exposure effects are also supported by a study of Dutch school children, where changes in play behavior among males and females in relation to PCBs were observed, with less masculine play behaviors among boys and more masculine play behaviors among girls (Vreugdenhil et al. 2002). Sex differences have also been reported in relation to other prenatal exposures, including lead, bisphenol A, and phthalates (Braun et al. 2009; Engel et al. 2010; Wasserman et al. 1998), supporting a growing belief that the neurodevelopmental impact of early-life exposure to environmental neurotoxicants on neurodevelopment is different for males and females (Weiss 2011).

Sexual dimorphism of effect has also been observed in animal studies in relation to PCB exposure, but results are inconsistent (Roegge et al. 2000; Schantz et al. 1995; Widholm et al. 2001). Although the biological basis for these sex-specific effects is not fully understood, prenatal PCB and dioxin exposure has been linked with gonadal hormone levels, including reduced cord serum testosterone levels in females and reduced estradiol levels in males (Cao et al. 2008). In addition, sex differences in prenatal gonadal hormones levels could explain differences in susceptibility to neurotoxicants, including PCBs (Seegal et al. 2010; Vahter et al. 2007).

Neuropsychological indicators of behavior, assessed in this study with the CPT and WISC-III, do not represent clinical diagnosis of ADHD but are neuropsychological correlates of ADHD that are measured on a continuum. Continuous outcomes are preferable for the purposes of research for three reasons: *a*) minimization of bias due to outcome misclassification, where categorization is often made at an arbitrary cutoff point that may change over time; *b*) detection of early or milder manifestations of a disorder that a clinical diagnosis could miss; and *c*) enhancement of power to detect an effect of an exposure (Bellinger 2004). In addition, these tests could be viewed as a more objective measure of functional limitations than parent or teacher report of observed behaviors.

However, a limitation of using the CPT and WISC-III outcomes as markers for inattentive and hyperactive/impulsive behaviors is that these outcomes may also reflect other skills that could compromise or enhance resulting scores for these neuropsychological tests. For example, longer mean reaction time could be a function of slower information processing skills, rather than, or in addition to, inattention—the confluence of which may be impossible to disentangle using a mean reaction time score.

A more conservative analytic approach may contribute to differences in our findings compared with other studies (Grandjean et al. 2001; Jacobson et al. 1992; Stewart et al. 2000, 2003, 2005). For example, CPT errors were modeled as count variables in this analysis, in contrast to previous studies that were not specific about how these errors were modeled but presumably used linear regression to model these outcomes (Jacobson and Jacobson 2003; Jacobson et al. 1992; Stewart et al. 2003, 2005). Given that count of errors (and their respective residuals) in this study did not follow a normal distribution and were overdispersed, the negative binomial distribution was a good fit for these data; this distribution is a conservative choice, however, as demonstrated by the wide CIs reported for CPT error outcomes. In previous studies using CPT error measures, model misspecification for count data may have led to underestimated variability of exposure effect estimates. We also omitted data for a child with an extremely high cord serum PCB level (ΣPCB_4_ > 4 ng/g); this data point disproportionately influenced the association between ΣPCB_4_ and errors of omission. After omitting data for this child, the association between ΣPCB_4_ and errors of omission was null for males and females combined, although it did persist for males (the child with omitted data is female).

## Conclusions

In summary, our results support an association between organochlorines, particularly PCBs, and neuropsychological measures of inattention. Our observation of an association among boys only has not been consistently explored in previous studies and warrants further exploration of sex-specific effects of organochlorines on these outcomes. These findings contribute to a growing literature showing associations between PCBs and ADHD-related behavior.

## Supplemental Material

(135 KB) PDF
