# Supplementary material for: Neuropsychological Measures of Attention and Impulse Control among 8-Year-Old Children Exposed Prenatally to Organochlorines
Source: Environ Health Perspect. 2012 Feb 22;120(6):904–9. doi: 10.1289/ehp.1104372 (PMC3385436; doi:10.1289/ehp.1104372)
Supplement: (135 KB) PDF [file ehp.1104372.s001.pdf]

SUPPLEMENTAL MATERIAL

Title: Neuropsychological Measures of Attention and Impulse Control Among 8-Year-Old Children Exposed Prenatally to Organochlorines

Authors: Sharon K. Sagiv[1,2], Sally W. Thurston[3], David C. Bellinger[4,5], Larisa M. Altshul[5,6], and Susan A. Korrick[2,5]

[1]Department of Environmental Health, Boston University School of Public Health, Boston, MA

[2]Channing Laboratory, Department of Medicine, Brigham and Women's Hospital, Boston, MA

[3]Department of Biostatistics and Computational Biology, University of Rochester School of Medicine and Dentistry, Rochester, NY

[4]Children's Hospital, Harvard Medical School, Boston, MA

[5]Department of Environmental Health, Harvard School of Public Health, Boston, MA

[6]Enviromental Health and Engineering, Needham, MA

Table of Contents

Supplemental Material, Table 1

Supplemental Material, Table 2

Supplemental Material, Table 1. Distribution of background characteristics and their unadjusted associations with CPT outcomes (n=578), for mothers and 8 year old children born in New Bedford, 1993-1998.

	No. (%)	Mean (SD)	Reaction Time (msec) β (95% CI)	Reaction Time Variability β (95% CI)	Errors of Omission RR (95% CI)	Errors of Commission RR (95% CI)
Parental characteristics						
Maternal age at child's birth (years)	578	26.7 (5.4)	0.35 (-0.64, 1.34)	-0.18 (-0.66, 0.30)	1.00 (0.98, 1.02)	1.00 (0.98, 1.02)
Maternal age category at child's birth (years)						
<20	76 (13.2)		-5.1 (-21.4, 11.2)	4.7 (-3.2, 12.7)	1.2 (0.9, 1.6)	1.0 (0.8, 1.3)
20-29	302 (52.3)		0	0	1.0	1.0
30-34	126 (21.8)		-0.7 (-14.2, 12.8)	1.8 (-4.8, 8.3)	1.0 (0.8, 1.3)	0.9 (0.7, 1.1)
35+	74 (12.8)		5.5 (-11.0, 22.0)	1.2 (-6.8, 9.3)	1.2 (0.9, 1.6)	1.0 (0.8, 1.3)
Maternal education at child's school age						
<12th grade	62 (10.8)		2.4 (-16.3, 21.1)	3.5 (-5.6, 12.6)	1.4 (1.0, 2.0)	1.3 (1.0, 1.6)
H.S. graduate	184 (32.2)		0	0	1.0	1.0
Some college	326 (57.0)		1.4 (-10.3, 13.1)	-2.5 (-8.2, 3.3)	0.9 (0.7, 1.1)	0.9 (0.7, 1.0)
Missing	6					
Paternal education at child's school age						
<12th grade	134 (24.4)		-8.8 (-22.5, 4.9)	-1.4 (-7.6, 4.7)	1.2 (0.9, 1.6)	1.0 (0.8, 1.2)
H.S. graduate	236 (43.0)		0	0	1.0	1.0
Some college	179 (32.6)		-7.7 (-20.3, 4.8)	-4.4 (-10.1, 1.2)	0.9 (0.7, 1.1)	0.9 (0.7, 1.0)
Missing	29					
Annual household income at child's school age						
<$20,000	116 (20.4)		11.1 (-2.9, 25.0)	11.9 (5.2, 18.6)	1.7 (1.3, 2.1)	1.4 (1.2, 1.7)
$20-39,999	161 (28.3)		-5.7 (-18.1, 6.8)	3.7 (-2.3, 9.8)	1.3 (1.0, 1.6)	1.1 (1.0, 1.4)
≥$40,000	293 (51.4)		0	0	1.0	1.0
Missing	8					
Maternal marital status at child's school age						
Married	340 (58.8)		0	0	1.0	1.0
Not married	238 (41.2)		-2.4 (-13.1, 8.4)	7.9 (2.7, 13.1)	1.3 (1.0, 1.6)	1.3 (1.2, 1.6)
Maternal smoking during pregnancy						
Yes	159 (29.6)		2.4 (-9.6, 14.4)	9.8 (4.0, 15.5)	1.1 (0.9, 1.4)	1.1 (0.9, 1.3)

No	378 (70.4)		0	0	1.0	1.0
Missing	41					
Maternal alcohol consumption during pregnancy						
<1 servings/month	444 (89.9)		0	0	1.0	1.0
1-2 servings/month	13 (2.6)		-29.9 (-65.5, 5.7)	-8.3 (-25.3, 8.8)	0.6 (0.3, 1.2)	1.1 (0.7, 1.8)
>2 servings/month	37 (7.5)		-2.9 (-24.5, 18.8)	-9.0 (-19.4, 1.3)	0.8 (0.6, 1.3)	0.9 (0.7, 1.3)
Missing	84					
Used illicit drugs in year prior to birth						
Yes	70 (14.2)		-1.0 (-17.4, 15.3)	2.6 (-5.2, 10.4)	1.1 (0.8, 1.4)	1.1 (0.8, 1.3)
No	422 (85.8)		0	0	1.0	1.0
Missing	86					
Maternal local fish consumption during pregnancy						
Yes	54 (10.9)		1.7 (-16.6, 20.0)	-0.5 (-9.3, 8.3)	0.9 (0.7, 1.3)	1.2 (0.9, 1.5)
No	440 (89.1)		0	0	1.0	1.0
Missing	84					
Maternal IQ[a]	575	97.9 (10.4)	-0.22 (-0.73, 0.30)	-0.35 (-0.60, -0.11)	0.98 (0.97, 0.99)	0.98 (0.98, 0.99)
Missing	3					
Maternal Depression[a]	576	8.4 (8.7)	0.09 (-0.51, 0.70)	0.25 (-0.05, 0.54)	1.01 (1.00, 1.02)	1.01 (1.00, 1.02)
Missing	2					
HOME score	564	45.6 (5.4)	-0.27 (-1.26, 0.72)	-0.86 (-1.35, -0.38)	0.97 (0.95, 0.99)	0.97 (0.96, 0.99)
Missing	14					
Child's characteristics						
Age at exam	578	8.2 (0.6)	-29.53 (-38.02, -21.05)	-0.94 (-5.24, 3.35)	0.83 (0.70, 0.98)	0.98 (0.87, 1.11)
Sex						
Male	294 (50.9)		-10.5 (-21.1, 0.1)	1.5 (-3.6, 6.7)	0.9 (0.8, 1.1)	1.2 (1.0, 1.4)
Female	284 (49.1)		0	0	1.0	1.0
Race/ethnicity						
White	391 (69.0)		0	0	1.0	1.0
Black	36 (6.4)		-14.2 (-36.4, 8.0)	-2.2 (-12.9, 8.6)	0.9 (0.6, 1.4)	1.0 (0.7, 1.4)
Hispanic	55 (9.7)		-6.4 (-24.7, 12.0)	-2.7 (-11.6, 6.1)	1.3 (1.0, 1.9)	1.2 (1.0, 1.6)

Cape Verdean	62 (10.9)	4.3 (-13.1, 21.8)	12.5 (4.1, 20.9)	1.3 (0.9, 1.7)	1.2 (0.9, 1.5)
Other	23 (4.1)	12.2 (-15.1, 39.6)	-6.8 (-20.0, 6.4)	1.5 (0.9, 2.5)	1.2 (0.8, 1.8)
Missing	11				
Breastfed					
Never	285 (49.5)	0	0	1.0	1.0
<1 month	82 (14.2)	0.7 (-15.1, 16.6)	-5.2 (-12.9, 2.6)	0.9 (0.7, 1.2)	1.0 (0.8, 1.3)
1-3 months	65 (11.3)	9.7 (-7.7, 27.1)	0.7 (-7.8, 9.1)	0.9 (0.6, 1.2)	0.9 (0.7, 1.1)
4-5 months	50 (8.7)	-3.8 (-23.2, 15.6)	-8.7 (-18.1, 0.8)	0.8 (0.6, 1.2)	1.0 (0.7, 1.3)
6+ months	94 (16.3)	21.7 (6.6, 36.7)	3.3 (-4.0, 10.6)	1.0 (0.8, 1.3)	1.0 (0.8, 1.2)
Missing	2				
Type of school					
Public	525 (90.8)	0	0	1.0	1.0
Private	53 (9.2)	0.2 (-18.2, 18.5)	0.2 (-8.7, 9.2)	0.8 (0.5, 1.1)	0.9 (0.7, 1.2)
Number of siblings living in house					
None	86 (14.9)	0	0	1.0	1.0
One	270 (46.7)	1.6 (-14.1, 17.4)	-8.4 (-16.0, -0.8)	0.8 (0.6, 1.1)	0.8 (0.7, 1.0)
Two or more	222 (38.4)	5.0 (-11.1, 21.2)	-11.4 (-19.2, -3.6)	1.0 (0.7, 1.3)	0.9 (0.7, 1.1)

Abbreviations: CPT=Continuous Performance Test.
[a] Maternal IQ was measured with the Kaufman Brief Intelligence Test and depression was measured with the Beck Depression Inventory.

Supplemental Material, Table 2. Distribution of background characteristics and their unadjusted associations with WISC-III outcomes (n=584), for mothers and 8 year old children born in New Bedford, 1993-1998.

	No. (%)	Mean (SD)	Processing Speed β (95% CI)	Freedom from Distractibility β (95% CI)
Parental characteristics				
Maternal age at child's birth (years)	584	26.7 (5.4)	0.49 (0.27, 0.71)	0.35 (0.15, 0.54)
Maternal age category at child's birth (years)				
<20	77 (13.2)		-4.6 (-8.2, -1.0)	-4.7 (-7.9, -1.4)
20-29	305 (52.2)		0	0
30-34	127 (21.8)		5.2 (2.2, 8.2)	1.5 (-1.2, 4.2)
35+	75 (12.8)		0.5 (-3.2, 4.1)	0.8 (-2.5, 4.1)
Maternal education at child's school age				
<12th grade	63 (10.9)		-7.7 (-11.8, -3.6)	-5.7 (-9.3, -2.0)
H.S. graduate	188 (32.5)		0	0
Some college	327 (56.6)		0.8 (-1.8, 3.3)	1.6 (-0.8, 3.9)
Missing	6			
Paternal education at child's school age				
<12th grade	134 (24.1)		-4.6 (-7.7, -1.6)	-3.5 (-6.2, -0.8)
H.S. graduate	241 (43.4)		0	0
Some college	180 (32.4)		2.3 (-0.5, 5.1)	3.9 (1.5, 6.4)
Missing	29			
Annual household income at child's school age				
<$20,000	118 (20.5)		-8.4 (-11.5, -5.3)	-6.4 (-9.2, -3.7)
$20-39,999	162 (28.1)		-4.3 (-7.0, -1.5)	-4.4 (-6.8, -1.9)
≥$40,000	296 (51.4)		0	0
Missing	8			
Maternal marital status at child's school age				
Married	342 (58.6)		0	0
Not married	242 (41.4)		-5.4 (-7.8, -3.0)	-3.6 (-5.7, -1.5)
Maternal smoking during pregnancy				
Yes	160 (29.5)		-5.0 (-7.7, -2.3)	-1.2 (-3.6, 1.1)

No	382 (70.5)		0	0
Missing	42			
Maternal alcohol consumption during pregnancy				
<1 servings/month	446 (89.6)		0	0
1-2 servings/month	14 (2.8)		-4.4 (-12.2, 3.3)	0.5 (-6.4, 7.3)
>2 servings/month	38 (7.6)		0.7 (-4.1, 5.6)	1.6 (-2.7, 5.8)
Missing	86			
Used illicit drugs in year prior to birth				
Yes	70 (14.1)		-3.4 (-7.1, 0.2)	-0.4 (-3.7, 2.9)
No	426 (85.9)		0	0
Missing	88			
Maternal local fish consumption during pregnancy				
Yes	54 (10.8)		-1.4 (-5.6, 2.7)	-0.6 (-4.2, 3.1)
No	444 (89.2)		0	0
Missing	86			
Maternal IQ[a]	581	97.9 (10.4)	0.25 (0.14, 0.37)	0.36 (0.26, 0.46)
Missing	3			
Maternal Depression[a]	582	8.4 (8.8)	-0.18 (-0.32, -0.05)	-0.15 (-0.27, -0.03)
Missing	2			
HOME score	570	45.6 (5.4)	0.50 (0.27, 0.72)	0.48 (0.28, 0.68)
Missing	14			
Child's characteristics				
Age at exam	584	8.2 (0.6)	-2.54 (-4.53, -0.56)	-0.76 (-2.53, 1.01)
Sex				
Male	299 (51.2)		-5.0 (-7.3, -2.6)	-0.2 (-2.4, 1.9)
Female	285 (48.8)		0	0
Race/ethnicity				
White	396 (69.1)		0	0
Black	36 (6.3)		-4.4 (-9.4, 0.5)	-0.4 (-4.9, 4.0)
Hispanic	55 (9.6)		-4.6 (-8.6, -0.5)	-4.6 (-8.3, -1.0)

Cape Verdean	62 (10.8)	-5.7 (-9.6, -1.9)	-2.6 (-6.1, 0.9)
Other	24 (4.2)	-2.1 (-8.1, 3.8)	-0.6 (-6.0, 4.9)
Missing	11		
Breastfed			
Never	288 (49.5)	0	0
<1 month	83 (14.3)	0.9 (-2.7, 4.5)	1.6 (-1.6, 4.7)
1-3 months	66 (11.3)	1.3 (-2.6, 5.2)	2.7 (-0.8, 6.2)
4-5 months	50 (8.6)	-1.1 (-5.5, 3.3)	-1.6 (-5.5, 2.3)
6+ months	95 (16.3)	0.2 (-3.2, 3.6)	0.9 (-2.1, 3.9)
Missing	2		
Type of school			
Public	530 (90.8)	0	0
Private	54 (9.3)	5.6 (1.5, 9.7)	4.1 (0.5, 7.8)
Number of siblings living in house			
None	88 (15.1)	0	0
One	273 (46.8)	1.1 (-2.5, 4.6)	-0.5 (-3.6, 2.7)
Two or more	223 (38.2)	-2.3 (-5.9, 1.3)	-1.8 (-5.0, 1.4)

Abbreviations: WISC-III= Wechsler Intelligence Test for Children – Third Edition.

[a] Maternal IQ was measured with the Kaufman Brief Intelligence Test and depression was measured with the Beck Depression Inventory.
